# Ventricular Fibrillation-Induced Cardiac Arrest Results in Regional Cardiac Injury Preferentially in Left Anterior Descending Coronary Artery Territory in Piglet Model

**DOI:** 10.1155/2016/5958196

**Published:** 2016-11-02

**Authors:** Giridhar Kaliki Venkata, John R. Forder, Dan Clark, Andre Shih, Sharda Udassi, Srinivasarao Badugu, Melissa A. Lamb, Stacy L. Porvasnik, Renata S. Shih, Dalia Colon-Lopez, Arno L. Zaritsky, Ikram U. Haque, Jai P. Udassi

**Affiliations:** ^1^Division of Pediatric Critical Care Medicine, Department of Pediatrics, University of Florida College of Medicine, Gainesville, FL, USA; ^2^Division of Pediatric Cardiology, Congenital Heart Center, Department of Pediatrics, University of Florida College of Medicine, Gainesville, FL, USA; ^3^Divisions of Radiology and Biomedical Engineering, University of Florida, Gainesville, FL, USA; ^4^Department of Anesthesiology, College of Veterinarian School, University of Florida, Gainesville, FL, USA; ^5^Division of Pediatric Hospitalist Medicine, Department of Pediatrics, University of Florida College of Medicine, Gainesville, FL, USA; ^6^Department of Pediatrics, Sidra Research and Medical Center, Doha, Qatar; ^7^Division of Pediatric Critical Care, Department of Pediatrics, Texas Tech University Health Sciences, El Paso Children's Hospital, El Paso, TX, USA; ^8^Department of Pediatrics, Children's Hospital of The King's Daughters, Eastern Virginia Medical School, Norfolk, VA, USA; ^9^Division of Pediatric Critical Care, Department of Pediatrics, University of Texas Health Science Center at Houston, Houston, TX, USA

## Abstract

*Objective*. Decreased cardiac function after resuscitation from cardiac arrest (CA) results from global ischemia of the myocardium. In the evolution of postarrest myocardial dysfunction, preferential involvement of any coronary arterial territory is not known. We hypothesized that there is no preferential involvement of any coronary artery during electrical induced ventricular fibrillation (VF) in piglet model.* Design*. Prospective, randomized controlled study.* Methods*. 12 piglets were randomized to baseline and electrical induced VF. After 5 min, the animals were resuscitated according to AHA PALS guidelines. After return of spontaneous circulation (ROSC), animals were observed for an additional 4 hours prior to cardiac MRI. Data (mean ± SD) was analyzed using unpaired* t*-test; *p* value ≤ 0.05 was considered statistically significant.* Results*. Segmental wall motion (mm; baseline versus postarrest group) in segment 7 (left anterior descending (LAD)) was 4.68 ± 0.54 versus 3.31 ± 0.64, *p* = 0.0026. In segment 13, it was 3.82 ± 0.96 versus 2.58 ± 0.82, *p* = 0.02. In segment 14, it was 2.42 ± 0.44 versus 1.29 ± 0.99, *p* = 0.028.* Conclusion*. Postarrest myocardial dysfunction resulted in segmental wall motion defects in the LAD territory. There were no perfusion defects in the involved segments.

## 1. Introduction

The incidence of pediatric cardiac arrest in United States is about 16,000 cases per year [1]. Despite efforts to improve the quality of cardiopulmonary resuscitation (CPR), the establishment of AHA CPR guidelines, and recommendations from the International Liaison Committee on Resuscitation (ILCOR) regarding CPR and emergency cardiovascular care, survival from in-hospital and out-of-hospital cardiac arrest in children remains poor.

In a prospective cohort study involving US and Canadian Resuscitation Outcomes Consortium (ROC) sites, the incidence of pediatric out-of-hospital cardiac arrest (OHCA) was 8.04 per 100 000 per person years and survival to hospital discharge among this population was 6.4% [2]. In-hospital cardiac arrest has better survival chances because of shorter delay in initiating resuscitation. Of the 2 studies that looked at in-hospital cardiac arrest, one reported incidence of in-hospital cardiac arrests to be 5.5% in children admitted to Pediatric Intensive Care Units (PICUs), out of which 62.7% achieved return of spontaneous circulation (ROSC) and 19.5% survived to hospital discharge [3]. Another study reported 3% incidence in cardiac arrest in all children admitted to Children's Institute in Sao Paulo, Brazil, during one year of the study. 64% attained ROSC and about one-third survived to hospital discharge [4].

Cardiac dysfunction and hypoxic brain injury are responsible for a greater degree of morbidity and mortality in the postarrest recovery period [5–8].

Cardiac dysfunction after ROSC clinically manifests as decreased contractility and as a global decrease in wall motion. This state of heart failure has been referred to as “global stunning,” “postresuscitation syndrome,” and “postarrest myocardial dysfunction.” Postarrest myocardial dysfunction is reversible and its onset, severity, and duration are directly related to the duration of cardiac arrest [9, 10]. Postresuscitation syndrome is a significant contributor of early morbidity and mortality, worsening to cardiogenic shock and, consequently, multiorgan dysfunction syndrome (MODS). In an adult retrospective study looking into the causes of cardiogenic shock, out-of-hospital cardiac arrest was the etiology in 31 of 459 patients. Despite being of small number compared to other groups, they had the highest rate of ECMO support. In addition, this particular group suffered 54.8% 7-day mortality and 74.2% 30-day mortality, which are twice as high compared to other etiologies of cardiogenic shock [11]. With such small number of patients surviving after being resuscitated in both pediatric and adult population, research into understanding the pathogenesis of postarrest myocardial dysfunction, its management, and prediction of mortality assumes utmost importance.

Postarrest myocardial dysfunction is multifactorial and there is no clearly defined pathogenesis. Ischemia, reperfusion injury, inflammation, and related cytokines, catecholamines, microvascular injury, and defibrillation individually or in combination cause postresuscitation myocardial failure [12–14].

Postarrest myocardial dysfunction manifests as uniform decrease in wall motion [14]. This was demonstrated by contrast ventriculography and transthoracic echocardiography in swine model. In all the published studies on swine postarrest myocardial dysfunction, VF was left untreated for 10 min and 15 min [9], 12 min [14], and 7 min [10]. In an early study that looked into functional and metabolic derangements in the myocardium after 4 min of untreated VF, wall motion defects were not studied [15]. In a study that compared the durations of untreated VF (4 min, 7 min, and 10 min) on diastolic dysfunction, there was 100% survival in 4-minute group and progressive worsening of diastolic function from 7 min to 10 min [16].

There are no studies to show if lesser duration of untreated VF would result in segmental wall motion defect in contrast to global and more severe dysfunction with VF of longer duration. Further, there is no evidence on how wall motion defects initially manifest and how they progress as duration of VF progresses. Hence, we chose 5 min of fibrillation-induced cardiac arrest, to be in between the spectrum of 100% survival with minimal dysfunction and worse diastolic function with global wall motion defects. Moreover, all these studies used echocardiography which may not define a regional wall motion defect as good as MRI (Magnet Resonance Imaging) does, used in our current study.

Global decrease in wall motion associated with postarrest myocardial dysfunction has been attributed to transient compromise of coronary perfusion [14]. Since this entails measuring coronary blood flow during cardiac arrest and resuscitation and is not technologically feasible, researchers relied on indirect evidence trying to look for frank myocardial infarction. Myocardial ischemia leading to an infarction and consequently regional wall motion defects requires no further questioning [17]. In addition, wall motion defects were shown to have high correlation with the duration of ischemia. There is a gap between the amount and duration of restriction of myocardial blood flow during cardiac arrest and resuscitation, wall motion abnormalities, and perfusion defects.

Wall motion was studied using contrast ventriculography and transthoracic echocardiograms in previously published literature [9]. Cardiac MRI is the gold standard in structural, functional, and perfusion analysis in humans. Its use in animals has also been validated for cardiovascular studies [18].

The aim of the study was to demonstrate if uniform global wall motion abnormality would manifest after ROSC from 5 min of untreated VF and to detect perfusion defects in areas of myocardial wall motion defects.

## 2. Materials and Methods

The study was approved by University of Florida Health Science Center Institutional Animal Care and Use Committee (IACUC) and conducted in accordance with its guidelines. Animals were randomized to two groups. The first group underwent baseline cardiac magnetoresonance imaging (MRI) and the second group underwent electrical induced VF leading to cardiac arrest followed by cardiopulmonary resuscitation (CPR) according to American Heart Association (AHA) guidelines. After achieving ROSC, there was a 4-hour observation, after which the animals underwent cardiac MRI.

### 2.1. Animal Preparation

Six-week-old (weight 15 ± 1.99 kg) farm piglets (University of Florida Swine Unit) of either sex were used in the study. The animals were sedated with intramuscular (IM) injection of Ketamine (15 mg/kg/dose). The level of sedation was maintained with 5% isoflurane in 100% oxygen delivered via nose cone. An intravenous access was obtained in the ear lobe. Oral endotracheal intubation was performed and depth of anesthesia was maintained with isoflurane between 1.5% and 3%. Ventilation was provided with rate- and volume-regulated ventilator (Surgivet Vaporstic Anesthesia Machine, Smiths Medical, USA). Continuous end-tidal carbon dioxide (CO_2_) was measured with inline end-tidal CO_2_ monitor (Nellcor). Rate and tidal volumes were adjusted to maintain end-tidal CO_2_ between 35 and 45 mmHg. Electrocardiographic (EKG) leads were placed and heart rate and rhythm were continuously monitored.

After getting vascular access and oral endotracheal intubation, the animals that were randomized to baseline cardiac MRI study underwent no further invasive monitoring. Only heart rate, oxygen saturations, respiratory rate, end-tidal CO_2_, and noninvasive blood pressure (NIBP) measurements were recorded.

Cutdowns were performed in the second group using sterile surgical technique to expose left internal jugular (IJ) vein, and femoral artery was accessed for invasive BP monitoring. A vascular introducer sheath (5 F and 15 cm) was placed in the left IJ and advanced to the junction of superior vena cava (SVC) and right atrium (RA). An 8 F catheter was placed in either of the femoral arteries for invasive blood pressure (BP) monitoring (fluid filled catheter transducer). A noncoated guide wire was passed into right ventricle via the sheath in left internal jugular vein. Its location within the right ventricle was confirmed by fluoroscopy. Alternating current was delivered into right ventricle via this guide wire to induce VF. Heparin (50 units/kg/dose) was given as a single bolus dose.

CPR was initiated after 5 min of untreated VF (no chest compression and no ventilation); CPR was performed in strict accordance with AHA Pediatric Advanced Life Support (PALS) guidelines. The same PALS certified provider performed chest compressions in all the experiments with a rate of 100/minute and 30 : 2 compressions to ventilation ratio. Defibrillation was attempted initially with 70 J biphasic current with subsequent shocks being 150 J. During the CPR, an epinephrine bolus 0.01 mg/kg (1 : 10,000) was given when necessary according to AHA guidelines. ROSC was defined after sustained palpable pulse and aortic systolic blood pressure greater than 60 mmHg for at least 1 min. After having achieved ROSC, the animals remained intubated and mechanically ventilated. Anesthesia was continued and the animals were monitored in the operating room for an additional 4 hours. All animals had cardiogenic shock secondary to postarrest myocardial dysfunction. Circulation was supported using epinephrine infusion. Function and perfusion studies were performed using 3-Tesla strength MRI. After data acquisition, the animals were humanely euthanized using euthanasia solution (Beuthanasia^R^).

### 2.2. MRI Protocol

Cardiac MRI was performed with the 3-Tesla Philips scanner, Achieva Whole Body MRI System (Philips Medical Systems, Best, Netherlands). Cine images were obtained by using turbo field echo sequences. Matrix position was 150 × 192. Other parameters for cine data are as follows: slice thickness 8 mm, contiguous slices, flip angle 15°, TR 5.33, echo 3.21, and minimum of 12 phases for each cardiac cycle were performed. Field of view was 77%  {dimensions being (1.21 × 150 = 181.5 mm), (1.21 × 192 = 232.32 mm), and (18 cm × 23 cm)} and the final resolution was 1.21 square mm.

Perfusion studies were done by steady state turbo field echo. Three slices were used one each at base, midwall, and apex. Matrix position was 61 × 104. Flip angle was 20°. Repetition time was 3.2 milliseconds and echo time was 1.6 milliseconds. Field of view was 1.25 × 61 = 76.25, 1.25 × 104 = 130, and 7.6 cm × 13 cm. Inversion time was 105 milliseconds.

### 2.3. Data Acquisition and Analysis

CAAS MRV (Magnetic Resonance Ventricular Analysis) research software (Pie Medical Imaging) was used to analyze the MRI data. Epicardium, endocardium, and papillary muscles were manually delineated. Heart segmentation was performed according to AHA guidelines [19]. 112 heart parameters were collected including 17-segment wall motion.

### 2.4. Statistical Analysis

Data are presented as mean ± SD (standard deviation) or absolute number and percentages. The data was analyzed using unpaired *t*-test. The *p* value of ≤0.05 was considered statistically significant in all tests. The analyses were performed using SPSS 13.0 software (SPSS, Chicago, IL). Interobserver variability was reduced by having 3 individuals, who are trained in CMR analysis, trace the myocardium during heart cycle, and analyze the data in a blinded fashion.

## 3. Results

### 3.1. Animal Characteristics

Twelve animals (6 per group) were randomized to baseline and cardiac arrest. Both groups did not have any statistical difference with respect to age (6 weeks), height, weight, and body surface area (BSA), as shown in Table 1. All animals survived to 4 hours after resuscitation and completion of MRI data acquisition.

### 3.2. Basic Hemodynamic Parameters

Heart rate (HR, beats/min) in the baseline group was 92.33 ± 8.52 compared to 118.33 ± 33.15 in the postarrest group (*p* = 0.03) (Table 2). This difference is attributed to epinephrine infusion that was administered at an average 0.12 mcg/kg/min to support the blood pressure in the cardiac arrest group secondary to cardiogenic shock. There was no difference in the rest of other hemodynamic data in the 2 groups. Ejection fraction (EF) in the baseline group was 46.43 ± 9.62 compared to 39.52 ± 13.51 in the postarrest group (*p* = 0.17). Stroke volume (SV, mL) in the baseline group was 18.34 ± 4.12 compared to 16.20 ± 5.16 in the postarrest group (*p* = 0.24). A similar trend was observed with respect to Stroke Volume Index (SVI), baseline 33.54 ± 6.64 compared to 31.35 ± 9.93 in postarrest (*p* = 0.35). Cardiac output (CO, L/min) was 1.67 ± 0.32 in the baseline group compared to 1.93 ± 0.92 in the postarrest group (*p* = 0.25) and cardiac index (CI, L/min/m^2^) was 3.06 ± 0.46 in baseline group compared to 3.75 ± 1.82 in the postarrest group (*p* = 0.19). The Left Ventricular End Diastolic Volume (LVEDV, mL) in the baseline group was 39.48 ± 3.36 compared to 43.02 ± 11.12 in the postarrest group (*p* = 0.23) and Left Ventricular End Diastolic Volume Index (LVEDVI) in the baseline group was 72.74 ± 7.49 compared to 83.16 ± 20.70 in the postarrest group (*p* = 0.13). Left Ventricular End Systolic Volume (LVESV, mL) in the baseline group was 21.13 ± 4.05 compared to 26.82 ± 11.59 in the postarrest group (*p* = 0.14). Left Ventricular End Systolic Volume Index (LVESVI) in the baseline group was 39.19 ± 9.27 compared to 51.81 ± 22.02 in the cardiac arrest group (*p* = 0.11).

### 3.3. Function Analysis

**Figure 1:**
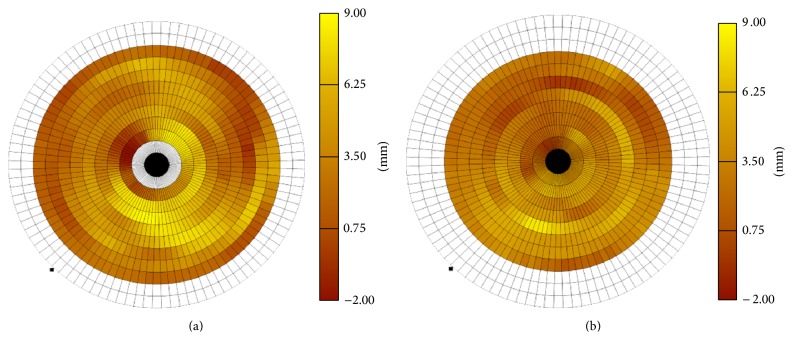
(a) Bull's eye representation of segmental wall motion score of baseline animal. (b) Bull's eye representation of segmental wall motion score of the same animal in Figure 1(a) but after resuscitation.

Segmental wall motion (mm) in segment 7 (midanterior, left anterior descending (LAD)) was 4.68 ± 0.54 in baseline group versus 3.31 ± 0.64 in postarrest group, *p* = 0.0026. In segment 13 (apical anterior, LAD), it was 3.82 ± 0.96 in baseline versus 2.58 ± 0.82 in postarrest, *p* = 0.02 (Table 3). In segment 14 (apical septal, LAD), it was 2.42 ± 0.44 in baseline versus 1.29 ± 0.99 in postarrest group, *p* = 0.028. The comparison of wall motion is shown in Figures 1(a) and 1(b). The first picture is a bull's eye representation of segmental wall motion score, which is color-coded according to the legend on the side of the picture. The second picture is a representation of the same animal after resuscitation; note that wall motion scores are much lower than previous bull's eye picture.

### 3.4. Perfusion Analysis

There was no difference in the perfusion parameters between baseline and postarrest myocardial dysfunction.

## 4. Discussion

Immediate mortality after resuscitation from cardiac arrest is primarily caused by myocardial dysfunction and hypoxic brain injury [8]. Postarrest myocardial dysfunction is manifested by decreased contractility and relaxation. On echocardiogram, it manifests as global wall motion defect [9]. There is an unexplored area between the extent and duration of myocardial blood flow restriction during cardiac arrest and resuscitation and the progression of wall motion abnormalities to a global dysfunction and finally cardiogenic shock.

Published studies involving swine on global decrease in wall motion reported resuscitation times after 7 min [10], 10 min, and 15 min of untreated cardiac arrest [9]. Animals with longer untreated cardiac arrest suffered from earlier manifestation of severe cardiogenic shock. In one study, hemodynamic parameters were studied after 4 min of untreated cardiac arrest, but wall motion was not studied [15]. Literature search revealed no studies on progression of wall motion defects from cardiac arrest through resuscitation and progression into postarrest phase. We are the first to report that, after 5 min of untreated cardiac arrest, segmental wall motion defects involved primarily the segments in left anterior descending (LAD) artery.

The animals that were studied in the cited references were of the age that ranged from 12 to 16 weeks [15]. In addition, the animals weighed 25 ± 2 Kg [14], 26 ± 1 Kg [9], 29 ± 1 Kg [20], 37 ± 2 Kg [21], 38 Kg to 45 Kg [16], and 40 ± 4 Kg [10]. We chose swine aged 6 weeks and weighed 15 Kg to replicate pediatric age group. Hence, we were unable to compare our hemodynamic data to previously published ones.

The coronary anatomy of swine is similar to that of humans with minor variations [22]. In majority of humans, right coronary artery is dominant [22]. Similar finding was reported in swine coronary artery distribution [22]. An important difference between the two coronary artery distributions is “Anterior Interventricular Vessel (AIV)” [22], which is LAD counterpart in swine, ends proximal to apex; in humans, it crosses the apex [22]. This is the reason for not observing wall motion defects in segment 17 (apex) in our swine model.

In all CPR experiments, chest compressions, defibrillation, and epinephrine are confounders to functional and perfusion data analysis.

Chest compressions have not been shown to cause localized wall motion abnormality. Although cardiac contusions were noted on necropsy in previously published studies, there were no transmural contusions. All of the contusions were localized to anterior portion of right ventricle, which does not happen to be our area of interest [9].

Defibrillation has been shown to be equivocal in causing myocardial dysfunction after resuscitation from cardiac arrest. However, in a study where defibrillation dose as high as 303 ± 38 J was tested without inducing cardiac arrest, there was no myocardial dysfunction [9].

The effect of epinephrine on segmental wall dyskinesis has not been reported.

In this experiment, we have established that wall motion defects are not uniform when resuscitated after 5 min of untreated VF. The defects were primarily present in the left anterior descending coronary distribution. It is our speculation that left ventricle is exposed to higher afterload and energy requirements and may be more susceptible to ischemic injury. In addition, we could not demonstrate any perfusion defects in segments 7, 13, and 14. Transient compromise in blood flow not to an extent to cause frank ischemia may have caused myocardial dysfunction. Our perfusion findings are in congruence with previously published reports of absence of ischemia in tissue sections [9]. The mechanisms underlying this dysfunction have not been conclusively defined. This remains an area of opportunity for further research.

Our study has several limitations. A shortcoming of our study is 5 min of cardiac arrest. Cardiac arrest was 10 min in one study that demonstrated uniform global wall motion defect [9]. Coronary artery anatomy was not studied prior to starting the experiments. Epinephrine was used to support hemodynamics in the animals that were resuscitated. Epinephrine helped to maintain cardiac output in these animals while MRI was being obtained. There was no statistical difference in ejection fraction in both groups due to use of epinephrine in the group which underwent resuscitation. Epinephrine might also have altered the wall motion findings. Our control group animals did not undergo CPR; an ideal control group would be to include animals that did not undergo cardiac arrest but received chest compressions and resuscitation. Since this experiment was terminal in nature, we could not establish the reversible nature of postarrest myocardial dysfunction.

## 5. Conclusion

Myocardial dysfunction following ventricular fibrillation-induced cardiac arrest results in nonuniform regional wall motion abnormalities especially in the left anterior descending artery territory. However, the abnormalities may not represent ischemia or infarction since perfusion studies did not reveal any defects.

Global ischemic insult resulted in segmental wall motion abnormality preferentially in left anterior descending artery territory. Higher heart rate and no significant difference in cardiac index were due to epinephrine infusion in cardiac arrest group.

## Figures and Tables

**Table 1:** Baseline animal characteristics.

Parameter	Baseline	Cardiac arrest	*p* value
*n*	6	6	n/a
Age, weeks	6	6	n/a
Weight, Kg	16.15 ± 2.23	13.88 ± 0.79	0.05
Length, m	0.76 ± 0.02	0.77 ± 0.01	0.37
BSA, Kg/m^2^	0.54 ± 0.03	0.51 ± 0.01	0.14

BSA, body surface area.

**Table 2:** Basic hemodynamic parameters.

Parameter	Baseline	Cardiac arrest	*p* value
Heart rate, bpm	92.33 ± 8.52	118.33 ± 33.15	0.03
Ejection fraction, %	46.43 ± 9.62	39.52 ± 13.51	0.17
Stroke volume, mL	18.34 ± 4.12	16.20 ± 5.16	0.24
Stroke Volume Index	33.54 ± 6.64	31.35 ± 9.93	0.35
LVEDV, mL	39.48 ± 3.36	43.02 ± 11.12	0.23
LVEDVI	72.74 ± 7.49	83.16 ± 20.70	0.13
LVESV, mL	21.13 ± 4.05	26.82 ± 11.59	0.14
LVESVI	39.19 ± 9.27	51.81 ± 22.02	0.11
CO, L/min	1.67 ± 0.32	1.93 ± 0.92	0.25
CI, L/min/m^2^	3.06 ± 0.46	3.75 ± 1.82	0.19

bpm: beats per minute; LVEDV: Left Ventricular End Diastolic Volume; LVEDVI: Left Ventricular End Diastolic Volume Index; LVESV: Left Ventricular End Systolic Volume; LVESVI: Left Ventricular End Systolic Volume Index; CO: cardiac output; CI: cardiac index.

**Table 3:** Segmental wall motion analysis (mm).

Parameters	Baseline	Cardiac arrest	*p* value
Segment 7	4.68 ± 0.54	3.31 ± 0.64	0.002
Segment 13	3.82 ± 0.96	2.58 ± 0.82	0.02
Segment 14	2.42 ± 0.44	1.29 ± 0.99	0.02
